# Otolaryngology – Head and Neck Surgeon unemployment in Canada: a cross-sectional survey of graduating Otolaryngology – Head and Neck Surgery residents

**DOI:** 10.1186/s40463-014-0037-3

**Published:** 2014-09-16

**Authors:** Michael G Brandt, Grace M Scott, Philip C Doyle, Robert H Ballagh

**Affiliations:** Department of Otolaryngology – Head and Neck Surgery, Division of Facial Plastic & Reconstructive Surgery, Faculty of Medicine, University of Toronto, Toronto, Ontario Canada; Rehabilitation Sciences, Faculty of Health Sciences, Western University, London, Ontario Canada; Department of Otolaryngology – Head and Neck Surgery, Schulich School of Medicine & Dentistry, Western University, London, Ontario Canada; Department of Surgery, Division of Otolaryngology, McMaster University, Hamilton, Ontario Canada

## Abstract

**Objective:**

Recently graduated Otolaryngology - Head and Neck Surgeons (OTO-HNS) are facing an employment crisis. To date, there has been no systematic evaluation of the factors contributing to this situation, graduating OTO-HNS trainee employment rates, nor the employment concerns of these graduating residents. This investigation sought to empirically evaluate prospective OTO-HNS graduate employment, identify factors contributing to this situation, and provide suggestions going forward.

**Methods:**

A cross-sectional survey of the 2014 graduating cohort of OTO-HNS residents was conducted 6-months prior to graduation, and immediately following residency graduation. Surveyed items focused on the demographics of the graduating cohort, their future training and employment plans, and their concerns relative to the OTO-HNS employment situation.

**Results:**

All twenty-nine Canadian medical school graduated OTO-HNS residents completed the initial survey, with 93% responding at the completion of residency. Only 6 (22%) indicated confirmed employment following residency training. 78% indicated that they were pursuing fellowship training. 90% identified the pursuit of fellowship training as a moderately influenced by limited job opportunities. The ability to find and secure full-time employment, losing technical skills if underemployed/unemployed, and being required to consider working in a less-desired city/province were most concerning. 34% of the residents felt that they were appropriately counseled during their residency training about employment. 90% felt that greater efforts should be made to proactively match residency-training positions to forecasted job opportunities.

**Conclusions:**

Canadian OTO-HN Surgeons lack confirmed employment, are choosing to pursue fellowship training to defer employment, and are facing startling levels of under- and unemployment. A multitude of factors have contributed to this situation and immediate action is required to rectify this slowly evolving catastrophe.

## Introduction

**Figure 1:**
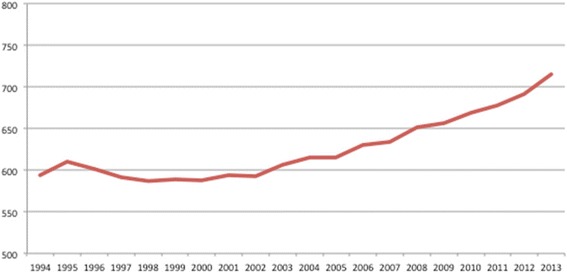
**Canadian Otolaryngology – Head and Neck Surgeons.** The number of Otolaryngology – Head and Neck Surgeons in Canada from 1994 to 2013.

Otolaryngology – Head and Neck Surgery (OTO-HNS) is a broad medical and surgical discipline that cares for numerous prevalent head and neck conditions in patients of all ages. Both community and academic Otolaryngology – Head and Neck Surgeons provide a vital role in supporting primary care practitioners in the care of their patients. Canada-wide there are approximately 715 OTO-HN Surgeons, reflecting 2.1 Surgeons per 100,000 people [1,2]. The number of OTO-HN Surgeons has been increasing steadily over the past 20-years (Figure 1). Between 1994 and 2004 the number of OTO-HN Surgeons increased from 594 to 615 (3.5% growth). The subsequent 10 years from 2004 to 2013, saw the number of OTO-HN Surgeons further increase from 615 to 715 – a 16% change. Table 1 demonstrates the age distribution of the 2013 cohort of Canadian OTO-HN Surgeons. 60% (n = 425) are below the age of 54 with the largest proportion under the age of 44 (n = 262 – 37%). Although 21% of OTO-HN Surgeons are above the age of 65, the retirement rate for the period 2010 to 2012 was 1.5% [1], suggesting a simple annual retirement rate of 0.5% or approximately 4 OTO-HN Surgeons per year. The overall youth of the specialty and the slow retirement rate are concerning when reviewed in the manpower planning context of the number of graduating residents and the number of graduates of Canadian medical schools (GCMS) entering the specialty.

**Table 1:** **Age of Otolaryngology – Head and Neck Surgeons in 2013**

**Age**	**n (%)**
≤ 44	262 (37%)
45 – 54	163 (23%)
55 – 64	124 (17%)
≥ 65	148 (21%)

[2] Data does not include 18 individuals who did not identify their age.

**Figure 2:**
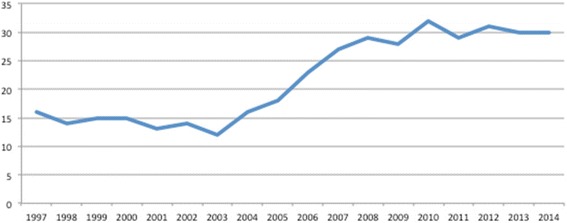
**Otolaryngology – Head and Neck Surgery R1 residency positions.** The number of first year Otolaryngology – Head and Neck Surgery positions available to graduates of Canadian medical schools from 1997 – 2013.

Each year, approximately 30 GCMS OTO-HN Surgeons graduate from OTO-HNS residency training programs. This number is reflective of a substantial increase in the volume of GCMS entering Canadian OTO-HNS residency training programs over the past 10 years (Figure 2) [3]. Prior to 2004, the average number of OTO-HNS residency positions available across Canada was 14.38 (13.40; 15.35 - [low; high] 95% CI) [3]. From 2004 to 2014, the number of first year OTO-HNS (R1) residency positions increased 93% to an average of 27.70 (25.09; 30.31) [3]. In the early 2000’s, a growing concern over a national physician shortage led to increased medical school training positions. As medical school spots increased in 2000 – 2002, a resultant increase in the number of all residency positions (including specialist positions) occurred in 2004 – 2006 to accommodate these new graduates. Surgical residencies including OTO-HNS also began to slowly increase the number of R1 residency positions. Interestingly, the number of OTO-HN Surgeons began to increase in 2004, preceding the trend expected from the increased number of OTO-HNS residency spots (an increase in spots in 2004 would impact the 2009 workforce due to the 5-year residency training). Although this curious finding precedes the increased number of residency positions, it appears to temporally relate to changes in American Board of Otolaryngology certification.

Otolaryngology – Head and Neck Surgeons graduating from residency prior to June 30^th^, 2005 (i.e. those “graduates of Canadian OTO-HNS programs entering residency training prior to July 1, 2000”) [4] are eligible for American Board of Otolaryngology (ABOTO) certification and thus immigration to the United States. Thus, in the years prior to 2005 OTO-HN Surgeons unable to obtain employment in Canada or choosing to immigrate to the United States were able to do so resulting in a buffer to the Canadian OTO-HNS workforce. While this number may have been limited (1 – 2 surgeons per year), restrictions on ABOTO certification would have resulted in an immediate, albeit minor, increase in the number of OTO-HN Surgeons in Canada. Expansion of residency training spots has thus further contributed to the number of Canadian OTO-HN Surgeons.

The multifactorial increase in the number of Canadian OTO-HN Surgeons has resulted in growing levels of under- and unemployment, morphed practices, and “brain waste” [5]. Unemployment is defined as a complete lack of gainful employment. Underemployment refers to part-time employment where full-time employment would be preferred and/or when individual education, skills, and training are not utilized to their full capacity. Morphed practices include the concept of combining multiple locum/part-time positions to work full-time hours, as well as, the alteration of one’s comprehensive skill-set to meet market needs (i.e. giving up a surgical practice in spite of being trained as a surgeon due to the absence of operative opportunities). Finally, “brain waste” occurs as a result of morphed practices and underemployment whereby skills (i.e. operative/technical skills) are lost due to underutilization. The 2013 Royal College of Physicians and Surgeons of Canada employment study suggested that 29% of graduating Otolaryngology – Head and Neck Surgeons are currently under- or unemployed, but the survey only had a 30% response rate [5]. Although the number of OTO-HN Surgeons who are currently under- or unemployed is unknown, the presence of any under- or unemployment amongst those dedicating nearly a decade of medical and surgical training is of great concern. As the number of OTO-HN Surgeons continues to increase, new OTO-HNS graduates face the prospect of an employment crisis. This investigation sought to capture the employment rate of the 2014 graduating OTO-HNS class, understand their future plans, and gain insight into the concerns they face in entering a saturated job market.

## Materials and methods

Ethics approval was obtained from the University of Western Ontario Research Ethics Board.

### Research protocol

All graduating OTO-HNS residents attending an annual OTO-HNS review course (January 13 – 18^th^, 2014) held in Halifax, Nova Scotia, Canada were invited to participate in this study. The goals of the investigation were announced to potential participants by one of the authors (MGB). Following the discussion of the goals of the investigation, a letter of information, consent form, and the investigative survey were distributed to all course attendees by a research assistant (GMS). Attendees were then voluntarily invited to participate in the completion of the research survey. Only the research assistant was present in the lecture theatre during survey distribution, completion, and collection and all course faculty were absent. All de-identified surveys were collected and retained by the research assistant, with data entry performed by the same assistant to ensure participant anonymity (Microsoft Excel, Microsoft Corporation, Redmond, WA, USA). Written feedback/comments that were provided by the survey participants were also confidentially collated and summarized by the research assistant (GMS). Given the small number of OTO-HNS residency programs and single province programs, participant geographic demographics were analyzed separately from survey responses to ensure complete participant anonymity and to maintain confidentiality. De-identified data summation and analysis were then performed by the investigative team (Microsoft Excel).

A secondary follow-up survey occurred in July 2014 at which time potential participants were emailed by the senior author (MGB). The email was sent as a batched/grouped email with recipient blinding to all potential participants of the aforementioned “Annual OTO-HNS review course”. No individual participant was recruited directly. Participants were asked to voluntarily email the senior author (MGB) to report on their employment situation at the completion of residency. They were specifically asked to report: “Employed - Community OTOHNS: Yes/No; Employed - Academic OTOHNS: Yes/No; Fellowship - Yes/No; If pursuing a fellowship, employment contract post fellowship - Yes/No”. These data were collected, de-identified, and subsequently anonymously collated by the senior author (MGB). All participant emails were then deleted to ensure confidentiality.

### Survey development

The investigative survey (Appendix A) was designed utilizing a group-consensus exercise. Items of investigative interest were discussed and summarized into survey questions which were then modified by the research team to ensure construct and content validity. Given the sensitive nature of the investigation and the requisite anonymity, no pilot testing was performed, nor were faculty at potential residency sites recruited to provide feedback relative to the survey.

### Statistical analysis

Descriptive statistics are presented as means with 95% confidence intervals as: mean ([min; max] 95% CI).

## Results

Twenty-nine postgraduate year five (PGY5) OTO-HNS residents completed the written survey in January 2014. This reflected 88% of the thirty-three 2014 graduating OTO-HNS trainees. The four non-participants were international medical graduates (IMG) who were asked to forgo study participation, thus, the present sample represents all (100%) of Canadian medical school graduates. Residents were on average 30 ± 2 years of age (Mean ± Standard Deviation), 76% (n = 22) were married, and 17% (n = 5) had children. The average estimated non-real-estate debt was assessed at approximately $100,000, with 34% (n = 10) carrying a debt-load greater than $100,000.

Of the 29 respondents, 5 (17%) indicated confirmed employment following residency training with 3 of these residents noting that their prospective job was contingent on the completion of a fellowship. Only 2 graduating residents noted confirmed employment specific to a community Otolaryngology – Head and Neck Surgery position; 83% of graduating residents did not have a confirmed job.

Of the 29 respondents, 18 were pursuing fellowship training (62%). A summary of fellowship training choices is presented in in Table 2. Of those surveyed, 90% indicated that their choice to pursue a fellowship was at least moderately importantly influenced by limited job opportunities in general/community OTO-HNS, and fellowship training offered an additional opportunity to defer entering the workforce (55% very important influence, 35% moderately important influence). Similarly, 42% of respondents indicated that if they pursued a fellowship they would very importantly increase their potential for obtaining a job (i.e., they perceived that a job opportunity would emerge in the area of their fellowship training), while 21% indicated that their desire to pursue fellowship training was unimportantly influenced by a potential job in that area. Sixty-seven percent (n = 14) of the residents pursuing fellowship training felt that the potential for private billings in the area of their fellowship training (i.e., non-provincially funded procedures such as balloon sinuplasty, professional voice clinics, cosmetic procedures, etc.) were unimportant in their fellowship decision.

**Table 2:** **Fellowship choice of the graduating 2014 OTO-HNS residents**

**Fellowship choice**	**Number (%)**
Head & Neck Oncology +/- Microvascular Reconstructive Surgery	6 (29%)
Laryngology	1 (5%)
Otology – Neurotology	6 (29%)
Rhinology +/- Anterior Skull Base Surgery	3 (14%)
Facial Plastic & Reconstructive Surgery	3 (14%)
Paediatric Otolaryngology	2 (10%)

**Figure 3:**
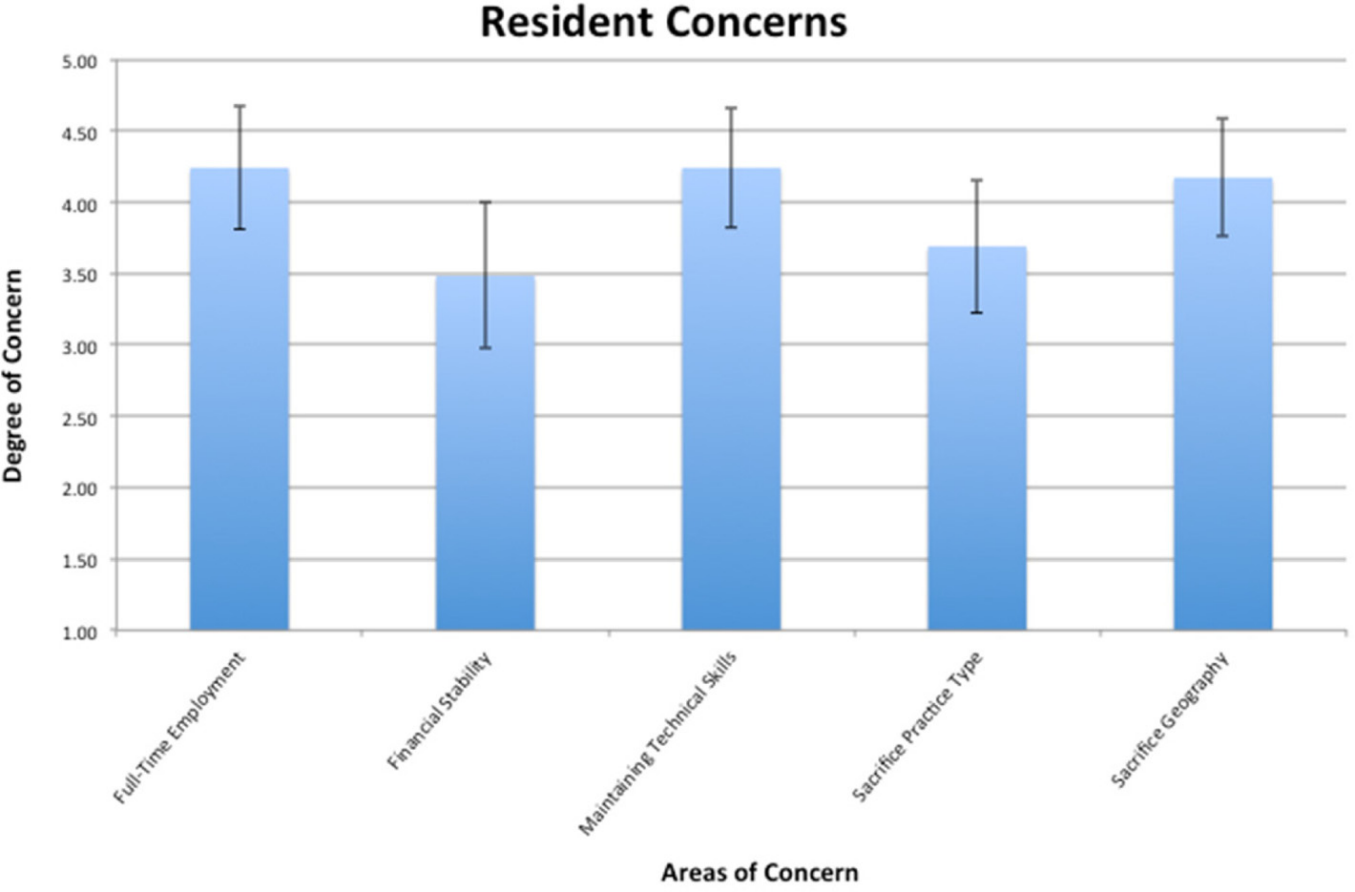
**Resident employment concerns.** Areas of concern appear on the x-axis, and degree of concern ranging from “Not at all concerned” (1) to “Very Concerned” (5) appear on the y-axis. Mean responses are illustrated with error bars representing 95% confidence intervals.

Residents universally identified a high degree of concern about the job market facing them upon graduating as OTO-HNS trainees (Figure 3). Potential responses ranged from 1 (“Not at All Concerned”) to 5 (“Very Concerned”). The ability to find and secure full-time employment (4.24 [3.81; 4.67] – mean [low; high] 95% CI), losing technical skills if underemployed/unemployed (4.24 [3.82; 4.66]), and being required to consider working in a less-desired city/province to obtain employment (4.17 [3.76; 4.59]) were of greatest concern. Further, being forced to sacrifice their professional desires (i.e., choosing a practice type they had not initially desired, for example, medical OTO-HNS in lieu of head and neck oncology) was also identified as being of considerable concern (3.69 [3.22; 4.16]). The residents’ ability to support themselves (including servicing their debt) was of moderate concern (3.48 [2.97; 3.99]).

In seeking additional information from respondents, 34% (n = 10) of the residents felt that they were appropriately counseled during their residency training about post-residency employment. However, 93% (n = 27) felt that their residency-training program should assist them in obtaining full-time employment; 90% (n = 26) of the graduating residents felt that greater efforts should be made to proactively match residency-training positions to forecasted job opportunities.

The final component of the survey asked respondents to provide comments on items not addressed in the survey. These comments were focused on several key areas: matching of OTO-HNS residency spots to forecasted need and succession planning (6), employment transparency (2), lack of United States “board eligibility” (2), the need for additional clinical and post-graduate academic training to obtain positions (2), and the use of residents and recent graduates to maintain clinician lifestyles (2). Comments that focused on matching residency spots to forecasted need directly described the conflict of few job opportunities paired with long patient wait-times, the high demand for OTO-HNS service, and the perceived and advancing age of current practitioners. Those concerned about employment transparency identified perceived circumstances where advertised jobs had already been filled or promised to others. At the present time Canadian Otolaryngology – Head and Neck Surgeons are not eligible for board certification in the United States, and respondents identified this as a real and significant problem that impeded their ability to apply their training. For this reason, respondents perceived this problem to result in a backlog of “skill-decaying qualified trainees”. Concerns referencing enhanced training focused on the need to obtain advanced degrees and fellowships to obtain employment relative to other medical colleagues and non-physicians. Finally, two respondents were concerned with the maintenance of current resident numbers as a means of imposing on-call and clinic-only (i.e. medical OTO-HNS) positions on new graduates to improve the quality of life for fully employed OTO-HN Surgeons (in both the academic and community setting).

The follow-up email survey in July 2014 demonstrated a 93% response rate (n = 27). Six participants indicated confirmed employment (22%) with 3 of these positions contingent on fellowship completion. Acknowledging non-respondents, these results show a single additional employed trainee from the initial January 2014 survey. Thus, 11% (n = 3) of the 2014 OTO-HNS cohort began practicing community OTO-HNS upon residency graduation. 11% (n = 3) of the participants reported that they were unemployed, reflecting a decrease from 6 respondents in the initial January survey. The number of participants pursuing fellowship training did however expand from 18 (62%) to 21 (78%).

## Discussion

Canada is facing a specialist under- and unemployment crisis. As the number of Otolaryngology – Head and Neck Surgeons expands, graduating OTO-HN Surgeons are increasingly forced to wrestle with both professional and personal compromises as they formally enter their careers. Although the number of underemployed OTO-HN Surgeons is unknown, the data presented herein suggest that 22% (n = 6) of the 2014 OTO-HNS graduating class have confirmed employment following residency or fellowship training, with only 11% (n = 3) entering the workforce immediately upon residency completion. Unlike the almost 40% of other graduating specialists [6] who would consider immigrating to the United States, Canadian OTO-HNS trainees are unable to do so which further escalates the rates of unemployment, underemployment, and brain waste. These results are alarming given the continued graduation of approximately 30 new OTO-HN Surgeons each year.

The tightening Canadian job market appears to be forcing specialists to pursue even further training. Not surprisingly, 78% (n = 21) of those surveyed indicated that they were pursuing a sub-specialty fellowship. This number is striking as it is more than double that of the entire cohort of Royal College of Physicians and Surgeons of Canada Specialists who when recently surveyed suggest only 31% were pursuing advanced training [5]. The economic impact of this decision cannot be discounted as over a third (34%) of the surveyed OTO-HNS residents indicated debt-loads greater than $100,000. Ninety percent (n = 19) of those surveyed identified fellowship training as a means of deferring entrance to the workforce – a salient comment vis-à-vis the current employment landscape, especially when considered in the context of the further accumulation of personal debt. Interestingly, fewer than half (42%) of the trainees indicated that fellowship training could very importantly influence their job prospects – indicating an awareness that in spite of advanced training, job opportunities may remain limited. This is further compounded by the OTO-HNS landscape whereby the majority of OTO-HN Surgeons work in a community setting and do not have sub-specialty practices. Thus, spending one to two-years of dedicated fellowship training away from general/community OTO-HNS may lead to an overproduction of subspecialists who are specialty focused and potentially uncomfortable with the breadth of community practice.

Predictably, the 2014 OTO-HNS graduating class identified securing full-time employment and losing technical skills as being of great concern. These findings align with concerns identified in both the National Physicians Survey and the Royal College employment survey [5,6]. Factors contributing to current specialist underemployment and the aforementioned concerns appear to stem from economic, systemic, and personal/contextual influences [5]. Hospital funding cutbacks have significantly affected resource intensive specialists. As OTO-HN Surgeons require operating room time and technical equipment, hospital cutbacks and resource limitations severely impact on OTO-HNS practice. With the economic downturn of 2008, tightening hospital budgets may be contributing to overall rates of OTO-HNS underemployment. This same economic downturn may also have impacted on OTO-HNS retirement planning contributing to the number of OTO-HN Surgeons currently practicing over the age of 65 (21%). Systemic factors that may be contributing to OTO-HNS under- and unemployment include the possible reliance on OTO-HNS residents and fellows within academic centres, poor workforce planning, restrictions from ABOTO certification, and the current culture of practice that encourages resource hoarding to protect patient wait-times. With the financial downturn in 2008, retirement delays were anticipated. In spite of this and the growing number of OTO-HN Surgeons, R1 resident positions continued to remain at an all-time high. Surgeons have long coveted their operating room time and hospital resources largely for the expedient provision of patient care. As such, Surgeons on the whole are reluctant to share resources to assist in the hiring of new graduates. Additionally, as OTO-HNS is a relatively small sector in the larger surgical demographic, any potentially available resources (i.e., planned retirement) is an opportunity for resources to be re-allocated to other surgical disciplines and those disciplines that potentially provide greater hospital funding. This type of strategy may again further exacerbate the situation that faces “smaller” surgical disciplines like OTO-HNS. Finally, the impact of personal factors cannot be discounted. Based on the present data, 76% (n = 22) of those surveyed were married and 17% (n = 5) had children. Sixty-six percent (n = 19) of the graduating OTO-HNS trainees also wished to practice in the province of their residency training. Suggesting that these individuals uproot their families without consideration of the potential impact on their established support network and a spouse’s employment is expectedly of great concern and is reinforced by our findings. Furthermore, pursuing subspecialty fellowship training in an area of interest irrespective of market demand may be contributing to under-employment and brain waste. Thus, the current situation of underemployment amongst OTO-HNS Surgeons appears to be multifactorial with contributions from economic, systemic, and personal factors.

The present data demonstrate a small number of employed graduating OTO-HNS residents and the substantial concerns these graduates face in entering the Canadian health care workforce. It is important to recognize that data from the initial and follow-up surveys are only reflective of employment conditions up to July 2014. Thus, employment rates identified herein may simply reflect the natural employment trend at that cross-sectional time-point and may not be indicative of true post-graduation employment. This is compounded by the absence of true under- and unemployment numbers amongst graduating OTO-HN Surgeons. Nevertheless, the trainees did identify substantial concerns over the employment landscape and its contributing factors. The existence of any involuntary under- or unemployment amongst Canadian OTO-HNS graduates is concerning and, therefor, as the number of OTO-HN Surgeons in Canada continues to expand and resource limitations remain the new normal, potential corrective strategies must be considered.

Careful, well-reasoned, and systematic action must be taken to improve the current employment situation. As a starting point, consideration must be made to decreasing the current number of OTO-HNS resident trainees to more accurately reflect the job market – a strategy supported by 90% (n = 21) of those surveyed. The present rate of graduation is unsustainable even with a mythical retirement of all OTO-HN Surgeons over the age of 65. The positions vacated through the retirement of the 148 OTO-HN Surgeons over the age of 65 would be saturated within 5 years by the current pipeline of OTO-HNS residents (5-years x ~30 OTOHN Surgeons per year). A possible barrier to decreasing residency-training spots is the necessity of residents to provide quality and efficient patient care in high-volume academic centres. As such, efforts must be made to expand the utilization of allied healthcare workers including nurse practitioners and physician assistants to maintain adequate patient care while ensuring that OTO-HNS resident trainees remain highly skilled and employable. Unfortunately, this strategy has a 5-year lag time. In the immediate future, a new focus must be directed toward transitioned retirements, protection of current resources, and very possibly the sharing of current resources. Introducing new graduates into established OTO-HNS practices could allow these graduates to maintain their skills, benefit from the mentorship of an experienced colleague, and ensure succession planning for busy community OTO-HNS practices. Those transitioning into retirement could benefit from decreased on-call responsibilities and the potential to bridge retirement through the provision of non-operative OTO-HNS care. This strategy could be implemented at a national level through the Canadian Society of Otolaryngology – Head and Neck Surgery (CSO-HNS) or more locally through academic training centres and their alumni network. The significance of the current employment problems and the substantial role OTO-HN Surgeons play in improving the quality of life of Canadians must be highlighted at a national level. While other areas of surgical care may provide improved reimbursement opportunities, Canadians rely on Otolaryngology – Head and Neck Surgeons and, thus, hospital resources must remain stable for the continued care of communities. To this end, hospital based OTO-HNS resources (i.e., OR time) must be viewed collectively rather than individually. OTO-HNS groups should ensure balanced and appropriate operating room resource allocation amongst all members of the group and consideration must be made to the potential of hiring new graduates should an abundance of resources exist. Finally, ABOTO eligibility as a buffer to Canadian employment should be considered. This would certainly improve current and immediate rates of under- and unemployment, but is largely a Band-Aid™ solution as workforce planning should ensure that investments in the training of Canadian OTO-HNS Surgeons are met with Canadian opportunities for these Surgeons.

In conclusion, the 2014 cohort of graduated Canadian Otolaryngology – Head and Neck Surgeons face real and substantial employment concerns and are choosing to pursue fellowship training to defer entering the job market. The potential for any involuntary under- and unemployment amongst those who have dedicated nearly a decade of their life to becoming OTO-HN Surgeons is unacceptable. A multitude of factors have contributed to this situation and immediate action is required to rectify this slowly evolving catastrophe.
